# Maternal sleep deprivation at different stages of pregnancy impairs the emotional and cognitive functions, and suppresses hippocampal long-term potentiation in the offspring rats

**DOI:** 10.1186/s13041-016-0197-3

**Published:** 2016-02-15

**Authors:** Yan Peng, Wei Wang, Tao Tan, Wenting He, Zhifang Dong, Yu Tian Wang, Huili Han

**Affiliations:** Ministry of Education Key Laboratory of Child Development and Disorders, Children’s Hospital of Chongqing Medical University, Chongqing, 400014 PR China; Chongqing Key Laboratory of Translational Medical Research in Cognitive Development and Learning and Memory Disorders, Children’s Hospital of Chongqing Medical University, Chongqing, 400014 PR China; Brain Research Centre and Department of Medicine, University of British Columbia, Vancouver, BC V6T 2B5 Canada

**Keywords:** Maternal sleep deprivation, Spatial learning and memory, Anxiety, Depression, Neurogenesis, Long-term potentiation

## Abstract

**Background:**

Sleep deprivation during pregnancy is a serious public health problem as it can affect the health of pregnant women and newborns. However, it is not well studied whether sleep deprivation at different stages of pregnancy has similar effects on emotional and cognitive functions of the offspring, and if so, the potential cellular mechanisms also remain poorly understood.

**Methods:**

In the present study, the pregnant rats were subjected to sleep deprivation for 6 h per day by gentle handling during the first (gestational days 1–7), second (gestational days 8–14) and third trimester (gestational days 15–21) of pregnancy, respectively. The emotional and cognitive functions as well as hippocampal long-term potentiation (LTP) were tested in the offspring rats (postnatal days 42-56).

**Results:**

The offspring displayed impaired hippocampal-dependent spatial learning and memory, and increased depressive- and anxiety-like behaviors. Quantification of BrdU-positive cells revealed that adult hippocampal neurogenesis was significantly reduced compared to control. Electrophysiological recording showed that maternal sleep deprivation impaired hippocampal CA1 LTP and reduced basal synaptic transmission, as reflected by a decrease in the frequency and amplitude of miniature excitatory postsynaptic current in the hippocampal CA1 pyramidal neurons.

**Conclusions:**

Taken together, these results suggest that maternal sleep deprivation at different stages of pregnancy disrupts the emotional and cognitive functions of the offspring that might be attributable to the suppression of hippocampal LTP and basal synaptic transmission.

## Background

Sleep is a basic necessity for survival, since long-term sleep deprivation leads to severe physical and cognitive impairment, even death [1]. However, about 50 % people throughout the world encounter one or more sleep disorders including insomnia, narcolepsy, somnambulism and the circadian rhythm sleep disorders [2]. Sleep disorders are especially prevalent in pregnancy due to a series of obvious reasons including pregnancy-associated hormonal, physical and behavioral changes [3, 4]. Accumulating evidence shows that sleep deprivation during pregnancy not only increases the risk of maternal psychiatric disorders [5], but also leads to several harmful consequences to the offspring. For example, maternal sleep deprivation impairs adult neurogenesis and hippocampus-dependent spatial learning and memory in the offspring rats [6, 7]. Furthermore, sleep deprivation during pregnancy leads to the lower sexual behavior of the male offspring [8] and increased risk-taking behavior in offspring [9, 10].

Although maternal sleep deprivation has been shown to cause behavioral alterations in offspring [6–11], there are few reports on the effects of sleep deprivation at different stages of pregnancy on emotional and cognitive functions of the offspring. And potential mechanisms underlying such effects also remain poorly understood. The hippocampal dentate gyrus plays a critical role in learning and memory throughout life, in part due to continuing neurogenesis occurrence in this area. There is a growing body of evidence showing that sleep deprivation reduces dentate gyrus neurogenesis, which may contribute to the deficit of hippocampal-dependent cognitive function [12–14]. However, the effects of sleep deprivation at different stages of pregnancy on adult hippocampal neurogenesis have not been extensively studied, although a recent report has showed that maternal sleep deprivation inhibits hippocampal neurogenesis in the young offspring rats [6]. In addition, several forms of synaptic plasticity in the CA1 area of the hippocampus, such as long-term potentiation (LTP) and long-term depression (LTD), have been proposed as the cellular mechanisms of information processing and memory formation [15–18]. Recent studies have demonstrated that sleep deprivation impairs hippocampal LTP [19, 20], which may be attributed to the disruption of cyclic adenosine monophosphate signaling [20]. However, there is no report about the influence of maternal sleep deprivation on hippocampal CA1 LTP in offspring.

Thus, the present study aimed to determine whether sleep deprivation at different stages of pregnancy affects emotional and cognitive functions of the offspring, and if so, whether this influence is related to adult hippocampal neurogenesis and hippocampal CA1 LTP in the offspring.

## Methods

### Animals

Female and male Sprague–Dawley rats were obtained from Chongqing Medical University Animal Care Centre, and they mated in the laboratory colony of Children’s Hospital of Chongqing Medical University. The offspring of these rats were used in the present study. Pregnant females were housed individually in plastic cages in the temperature-controlled (21 °C) colony room on a 12/12 h light/dark cycle (8:00 a.m.–8:00 p.m.), and were free to food and water. The pregnant rats were divided into 4 groups: normal rearing (control), sleep deprivation at early pregnancy stage (ESD, gestational days 1–7), sleep deprivation at middle pregnancy stage (MSD, gestational days 8–14) and sleep deprivation at late pregnancy stage (LSD, gestational days 15–21). Within 24 h of birth, all litters were culled to 10 pups with a goal of balancing the number of males and females equally. Young adult rats from postnatal day (PND) 42–56 were used for behavioral and electrophysiological experiments. All experimental protocols were approved by Chongqing Medical University Animal Care Committee.

### Sleep deprivation

The sleep deprivation was performed by gentle handling for 6 h per day (12:00–18:00) as previously described [10, 20]. Briefly, pregnant rats were kept awake by gentle tapping or rattling of the cage and, if necessary, by gently being touched with a soft brush if behavioral signs of sleep were observed, such as closed eyes and immobility. Food and water were available *ad libitum* throughout the sleep deprivation period.

### Reagents and antibodies

All drugs were purchased from Sigma-Aldrich. Mouse Anti-BrdU monoclonal antibody was purchased from Sigma-Aldrich. Rabbit anti-NeuN monoclonal antibody was purchased from Millipore. Rabbit anti-GFAP monoclonal was purchased from Abcam. Complete protease inhibitor cocktail tablets and phosphatase inhibitor cocktail tablets were purchased from Roche Applied Science.

### Morris water maze

Spatial learning and memory were examined with the Morris water maze using programs similar to those described previously [21, 22]. Briefly, rats were trained in a circular fiberglass pool (180-cm diameter) over 4 trials per day for 6 consecutive days to find a hidden platform. During each trial, the rats that cannot find the hidden platform within 60 s were guided to the platform where they remained for 20 s. A probe test was performed 24 h after the last learning trial. All trials were recorded and analyzed by using an Any-maze tracking system (Stoelting, USA).

### Elevated plus maze test

The plus maze apparatus consisted of two opposite open arms and two opposite closed arms (20-cm-tall walls on the closed arms) arranged at right angles. At the beginning of the test, rats were put in the center of the apparatus, which is elevated 1 m above the floor. The number of entries and the time spent in each arm were recorded for 10 min by ANY-maze video tracking system.

### Novelty-suppressed feeding test

Novelty-suppressed feeding test was performed as described previously [23]. In brief, rats were deprived of food for 48 h prior to the test. During test, a single pellet of food was placed on a white filter paper located in the center of the arena (60 × 60 cm), and rat was placed in a corner of the arena to explore the arena for 12 min. The latency to begin eating food and the amount of food consumption were recorded. The rats were immediately returned to their home cages after test, where food consumption was monitored for another 30 min.

### Forced swimming test

Rats were forced to swim in a cylinder filled with water (temperature 24–25 °C; 20 cm in diameter, 40 cm in height) for 10 min. The latency to immobility and total immobility time were recorded and analyzed by using ANY-maze video tracking system.

### Immunohistochemistry

To label newborn cells, rats were subjected to BrdU injection (100 mg/kg, i.p.) at age of 2 weeks or 6 weeks, and were sacrificed 4 weeks or 24 h after the last BrdU injection, respectively. The animals were deeply anesthetized and transcardially perfused with 4 % paraformaldehyde in 100 mM phosphate buffer, pH 7.4. Immunohistochemistry was performed on 30-μm coronal sections as previously described [23, 24]. Every sixth slice with the same reference position was stained. Positive cells were quantitated using a 40× objective (Leica). Obtaining numbers were multiplied by 6 to determine the estimated total number of positive cells per dentate gyrus (DG) of rat.

### Electrophysiology in vivo

Rats were deeply anesthetized with sodium pentobarbital at a dose of 60 mg/kg (i.p.) and then mounted to a stereotaxic frame (Stoelting Co.). The core temperature was monitored and kept at 36.5 °C ± 0.5 °C. Stimulating and recording electrodes (a pair of 100 μm outer diameter Teflon-coated wires; A-M Systems Inc.) were located at the Schaffer collaterals of dorsal hippocampus and ipsilateral striatum radiatum of hippocampal CA1 area, respectively. Final positions of the electrodes were determined when an optimal response of field excitatory post-synaptic potential (fEPSP) was obtained. Baseline responses were recorded at 0.033 Hz for 30 min, with an intensity that evoked half of maximum amplitude. Once the stable baseline was obtained, a HFS consisted of 100 pulses at 100 Hz was delivered to induce LTP.

### Electrophysiology in vitro

Rats were deeply anesthetized with 25 % urethane (1.5 g/kg, i.p.) and transcardially perfused with NMDG artificial cerebral spinal fluid (in mM: NMDG 93, HCl 93, KCl 2.5, NaH_2_PO_4_ 1.2, CaCl_2_ 0.5, MgSO_4_ 10, NaHCO_3_ 30, HEPES 20, Na-ascorbate 5.0, Na-pyruvate 3.0, Thiourea 2.0, NAC 12, and D-glucose 25, pH = 7.3.) prior to decapitation. The brain was rapidly dissected and placed in ice-cold NMDG ACSF. Hippocampal slices (400 μm) were coronally sectioned with a vibratome (VT1200S, Leica Microsystems, Bannockburn, IL) and then were incubated in HEPES ACSF (in mM: NaCl 92, KCl 2.5, NaH_2_PO_4_ 1.2, CaCl_2_ 0.5, MgSO_4_ 10, NaHCO_3_ 30, HEPES 20, Na-ascorbate 5.0, Na-pyruvate 3.0, Thiourea 2.0, NAC 12, and 25 D-glucose, pH = 7.3.) for 1 h at 30 °C.

Miniature excitatory postsynaptic currents (mEPSCs) of hippocampal CA1 pyramidal neurons were recorded with pipette filled with internal solution (in mM: Cs-methanesulfonate 130, MgCl_2_ 2.0, EGTA 0.5, HEPES 10, QX-314 5.0, K_2_ATP 5.0, and Na_2_GTP 0.3, pH = 7.3), resistance of which was 3–5 MΩ. Bicuculline methiodide (10 μM) and TTX (1 μM) were added in ACSF (in mM: NaCl 120, KCl 2.5, NaH_2_PO_4_ 1.25, CaCl_2_ 2.0, MgSO_4_ 2.0, NaHCO_3_ 26, glucose 10, pH = 7.3) to block GABA receptors and Na^+^ channels respectively. Data acquisition (filtered at 3 kHz and digitized at 10 kHz) was performed with PatchMaster v2.73 (HEKA Electronic, Lambrecht/Pfalz, Germany) with holding potential at −70 mV. Mini Analysis Program 6.0.3 (Synaptosoft Inc., Decatur, GA) was used to automatically detected mEPSCs.

### Statistical analysis

All data are presented as mean ± SEM. Spatial learning data were analyzed by a two-way ANOVA, with treatment (group) as the between-subjects factor and learning day as the within-subjects factor. All the other data were analyzed by a one-way ANOVA, with treatment (group) as the between-subjects factor. Significance level was set at *p* < 0.05.

## Results

### Maternal sleep deprivation impairs spatial learning and memory in the offspring rats

**Fig. 1:**
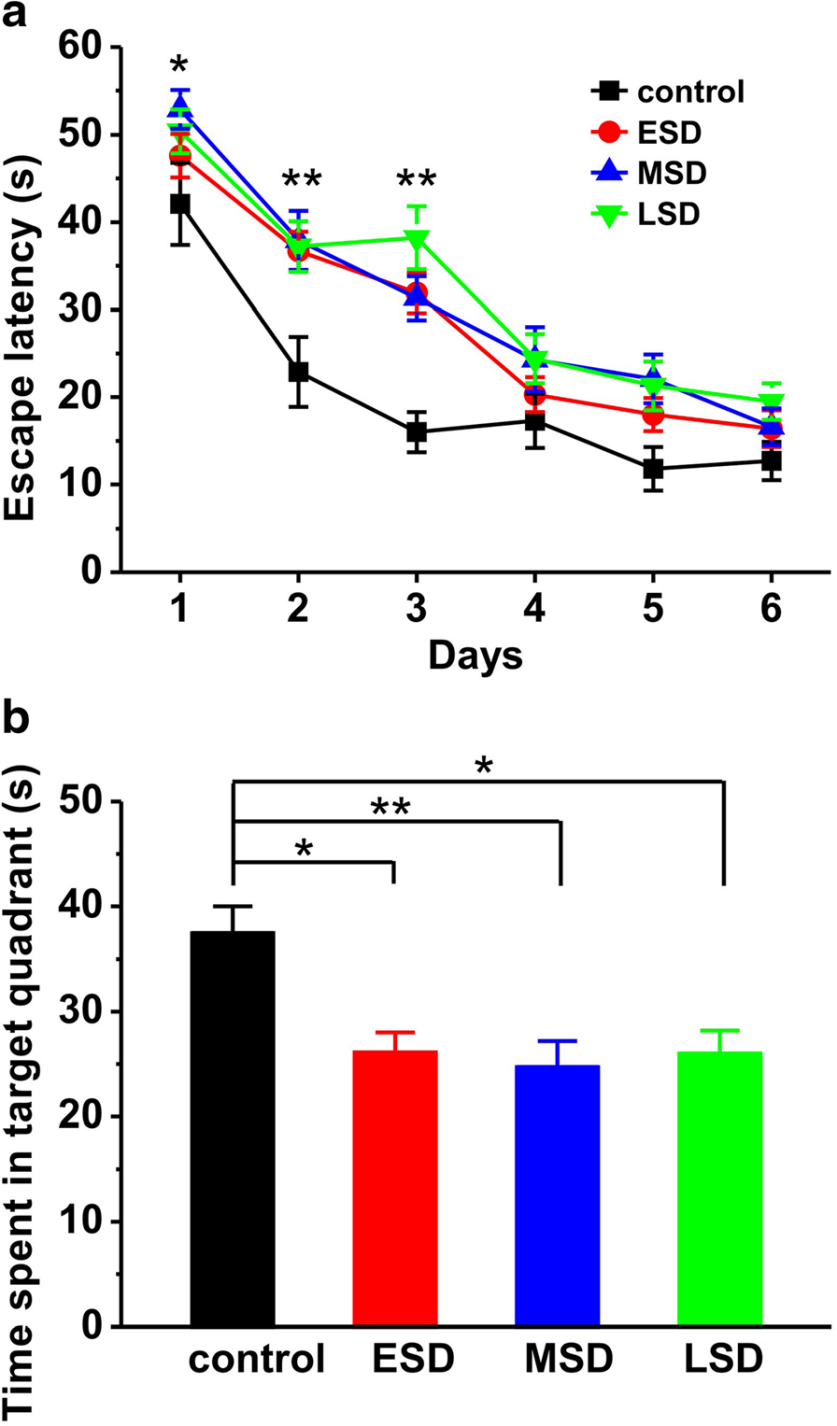
Maternal sleep deprivation impaired spatial learning and memory in the Morris water maze task. **a** The offspring of ESD (*n* = 16), MSD (*n* = 16) and LSD (*n* = 16) spent much longer time than control (*n* = 8) in searching the hidden platform on training day 1–3. **b** A probe test was performed on day 7, the offspring of ESD, MSD and LSD spent much less time than control in the quadrant where the the hidden platform was located. ^*^ *p* < 0.05, ^**^ *p* < 0.01 vs. control

We first determined the influence of sleep deprivation at different stages of pregnancy on spatial learning and memory in the young adult offspring. The pregnant rats were subjected to ESD, MSD and LSD, respectively. Spatial learning and memory was assessed using the Morris water maze in the offspring on PND 42–56. The offspring rats of all maternal sleep deprivation groups displayed a significant deficit in spatial learning, as reflected by taking much longer to find the hidden platform than control on day 1–3 (day 1: F _(3, 52)_ = 2.825, *p* = 0.048; day 2: F _(3, 52)_ = 4.370, *p* = 0.008; day 3: F _(3, 52)_ = 7.320, *p* < 0.001; Fig. 1a). One day after the last training trial, a probe test with the platform removed was performed to examine long-term spatial memory retrieval. The results revealed that maternal sleep deprivation dramatically impaired spatial memory retrieval in the offspring since they spent much less time in the quadrant in which the platform was previously located (F _(3, 52)_ = 4.343, *p* = 0.008; Fig. 1b). These results suggest that maternal sleep deprivation at different stages of pregnancy displays similar deficits in spatial learning and memory.

### Maternal sleep deprivation increases anxiety and depressive behaviors in the offspring rats

**Fig. 2:**
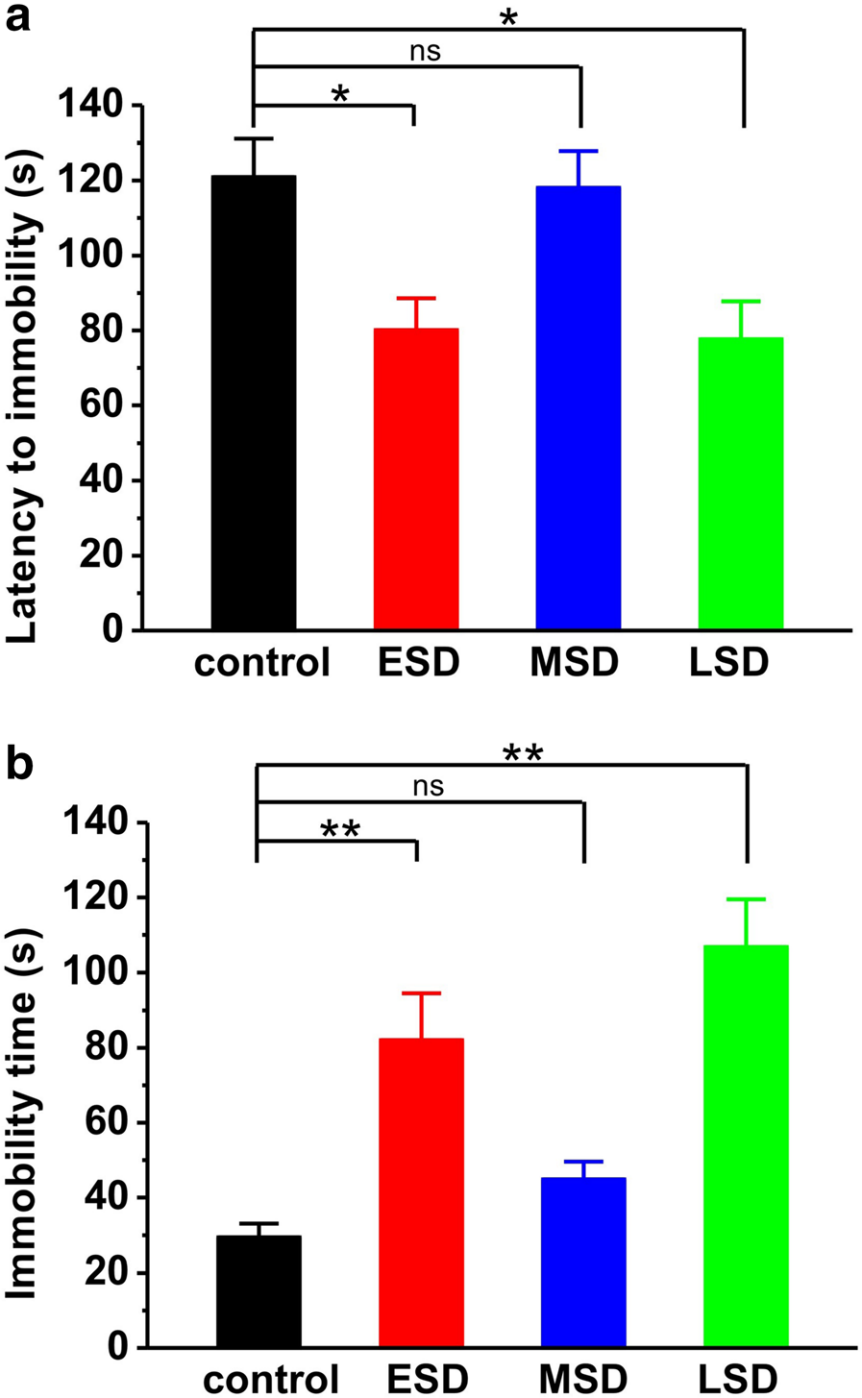
Maternal sleep deprivation increased depressive-like behavior in the forced swimming task. **a** The offspring of ESD (*n* = 12) and LSD (*n* = 20), but not MSD (*n* = 18), showed significantly shorter latency to immobility than control (*n* = 18) during forced swimming test. **b** The duration of total immobility was much longer in the offspring of ESD and LSD, but not MSD, than control. ^*^ *p* < 0.05, ^**^ *p* < 0.01 vs. control; ns = no significant difference

Next, we wanted to determine the influences of maternal sleep deprivation on emotional functions such as depression and anxiety in the offspring. The results showed that the latency to immobility was significantly shorter in the offspring of ESD and LSD, but not MSD, compared to the control rats during forced swimming test (F _(3, 64)_ = 5.578, *p* = 0.002; Fig. 2a). To further characterize the increase in depression, we also examined the total immobility time and found that the total immobility time was significantly longer in the offspring of ESD and LSD than other groups (F _(3, 64)_ = 15.669, *p* < 0.001; Fig. 2b).

**Fig. 3:**
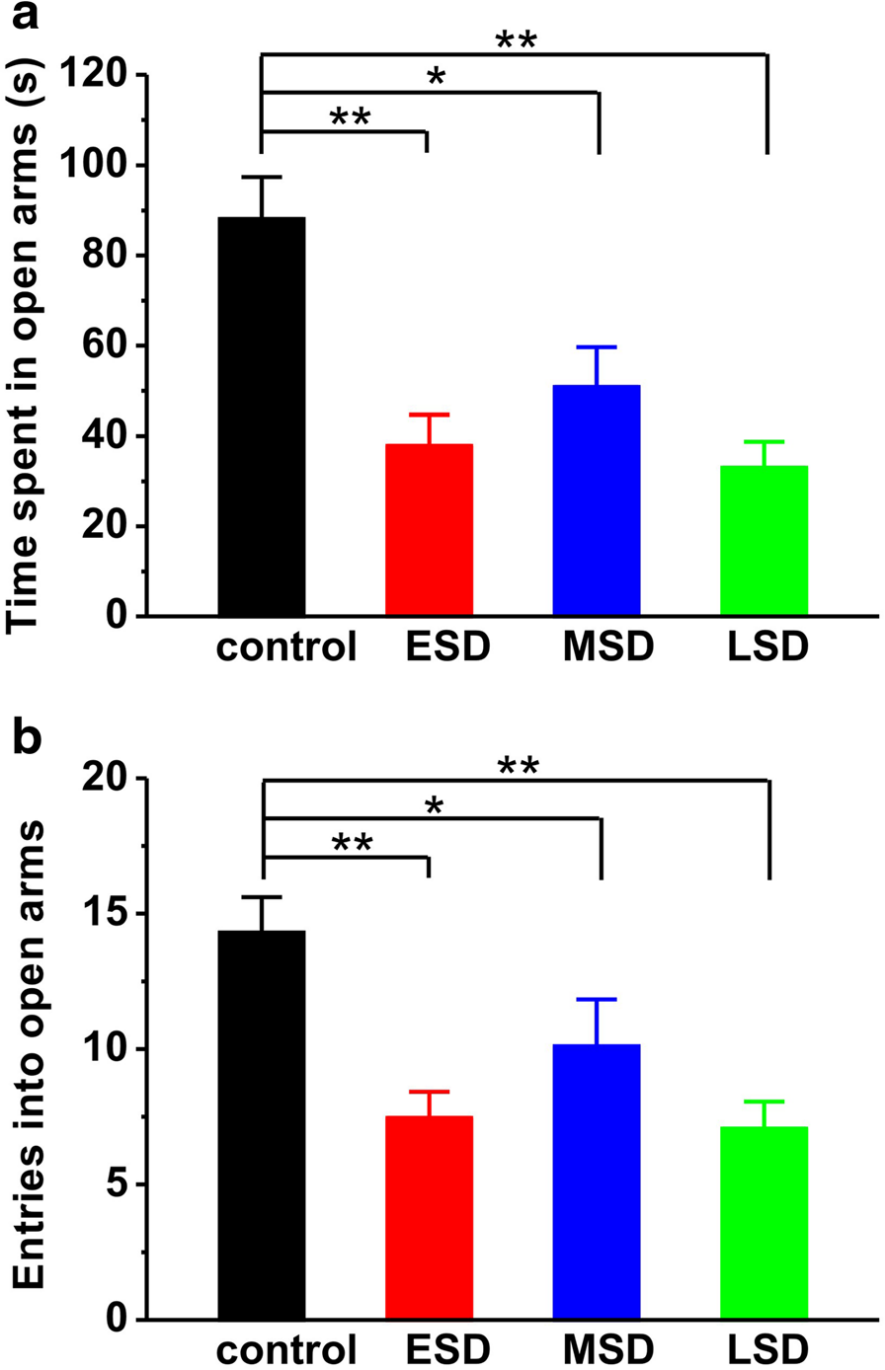
Maternal sleep deprivation increased anxiety-like behavior in the elevated plus maze task. **a** The offspring of ESD (*n* = 22), MSD (*n* = 18) and LSD (*n* = 21) spent much less time than control (*n* = 24) in the open arms. **b** The number of entry into open arms was significantly reduced in the offspring of ESD, MSD and LSD compared to control. ^*^ *p* < 0.05, ^**^ *p* < 0.01 vs. control

**Fig. 4:**
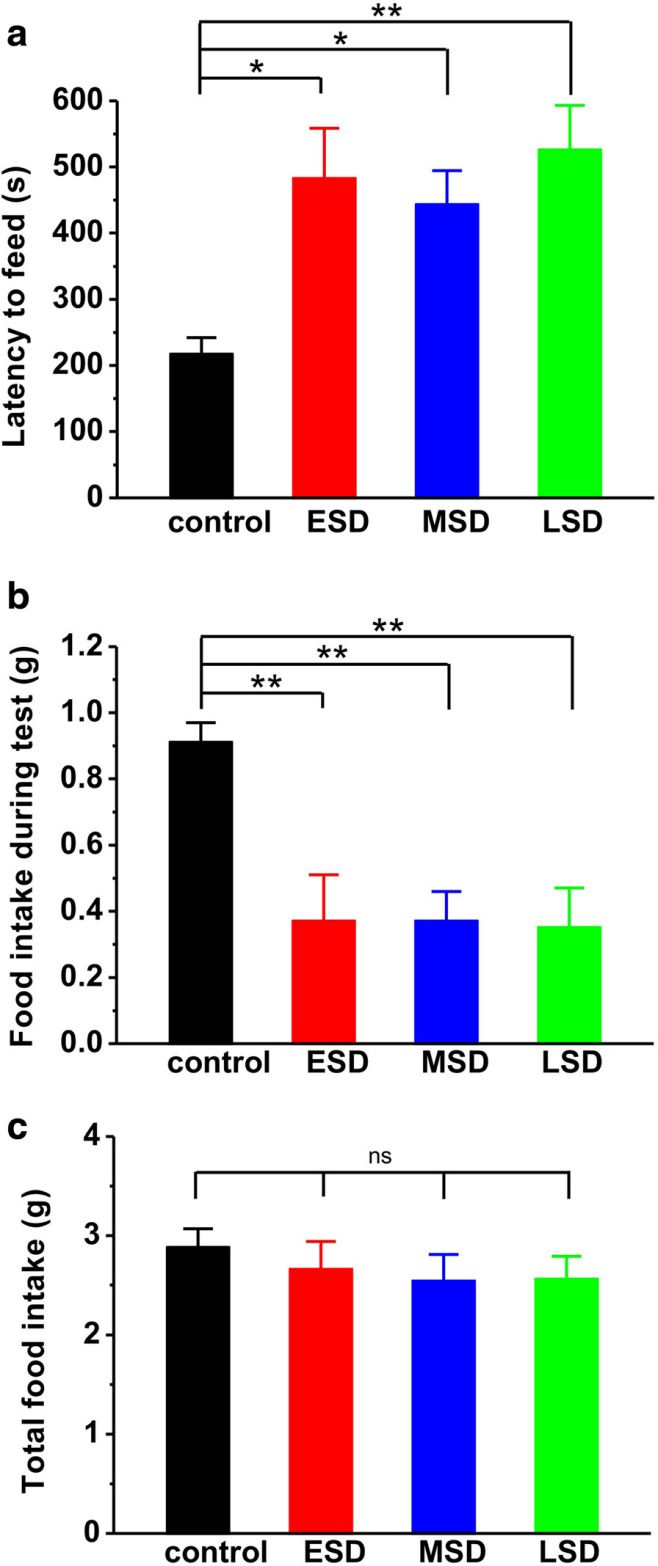
Maternal sleep deprivation increased anxiety-like behavior in the novelty-suppressed feeding task. **a** The offspring of ESD (*n* = 12), MSD (*n* = 12) and LSD (*n* = 12) showed significantly longer latency to feeding than control (*n* = 12) during the test. **b** The offspring of ESD, MSD and LSD consumed less food than control during the test. **c** No differences were observed in total food consumption including during test and in home cage among these groups. ^*^ *p* < 0.05, ^**^ *p* < 0.01 vs. control; ns = no significant difference

In Fig. 3, the results showed that both the time spent in the open arms (F _(3, 81)_ = 9.990, *p* < 0.001; Fig. 3a) and the number of entry into the open arms (F _(3, 81)_ = 7.187, *p* < 0.001; Fig. 3b) were significantly reduced during elevated plus maze test in offspring rats of ESD, MSD and LSD compared with control. In order to further evaluate the effect of maternal sleep deprivation on anxiety, we introduced another behavioral model of anxiety, the novelty-suppressed feeding test. The result showed that the latency to feeding was dramatically increased (F _(3, 44)_ = 5.534, *p* = 0.003; Fig. 4a), whereas the amount of food consumed was significantly reduced in the offspring rats of ESD, MSD and LSD during test (F _(3, 44)_ = 6.544, *p* = 0.001; Fig. 4b). These differences were not attributed to the influence of maternal sleep deprivation on the appetite in the offspring as the total food consumption, including food intake during test and in the homecage, remained unchanged among these groups (F _(3, 44)_ = 0.403, *p* = 0.752; Fig. 4c).

Taken together, these results indicate that maternal sleep deprivation increases anxiety and depression in the offspring rats.

### Maternal sleep deprivation reduces adult hippocampal neurogenesis in the offspring rats

**Fig. 5:**
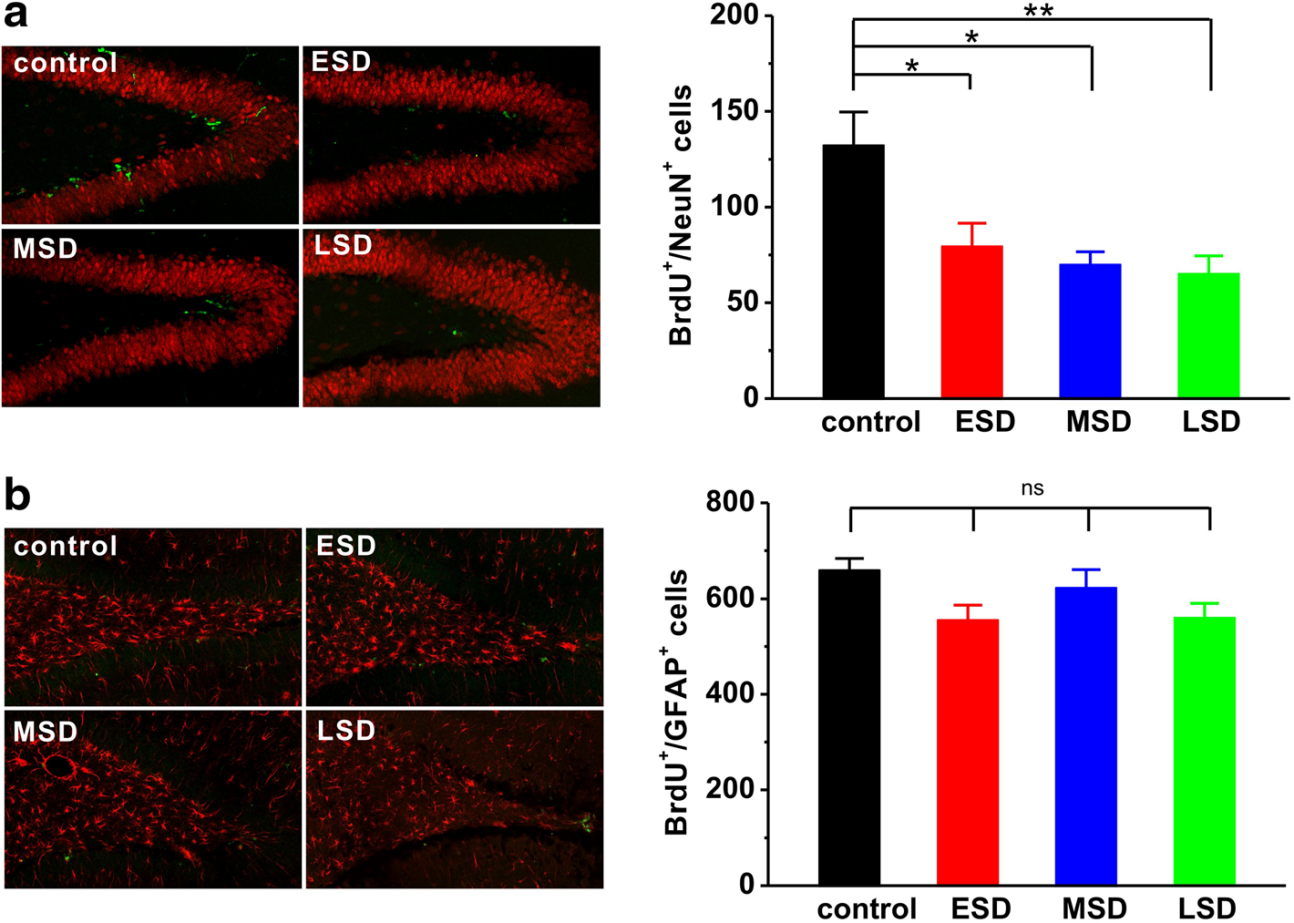
Maternal sleep deprivation reduced adult hippocampal neuronal proliferation. **a** The offspring of ESD (*n* = 5), MSD (*n* = 5) and LSD (*n* = 5) showed significantly reduced the immunoreactivity (left panel) and number of newborn neuronal phenotype cells (right bar) compared to control (*n* = 5), which were counted 24 h after last BrdU administration. **b** No differences were observed in the immunoreactivity (left panel) and number of newborn astrocytic phenotype cells (right bar) among these groups. ^*^ *p* < 0.05, ^**^ *p* < 0.01 vs. control; ns = no significant difference

We next tested the effects of sleep deprivation at different stages of pregnancy on hippocampal neurogenesis in the offspring. To observe cell proliferation in the dentate gyrus of the hippocampal formation, we treated animals with BrdU daily from PND 42–44, and sacrificed them at PND 45. Since different phenotypes of the proliferated cells can be labeled by BrdU, we used immunofluorescent double-labelings of brain sections with BrdU and either a neuronal (NeuN) or an astrocyte (GFAP) marker. We found that the number of BrdU-positive cells with neuronal phenotype (F _(3, 16)_ = 6.620, *p* = 0.005; Fig. 5a) rather than astrocytic phenotype (F _(3, 16)_ = 2.976, *p* = 0.063; Fig. 5b) was significantly reduced in the offspring of all maternal sleep deprivation groups compared to the control rats.

**Fig. 6:**
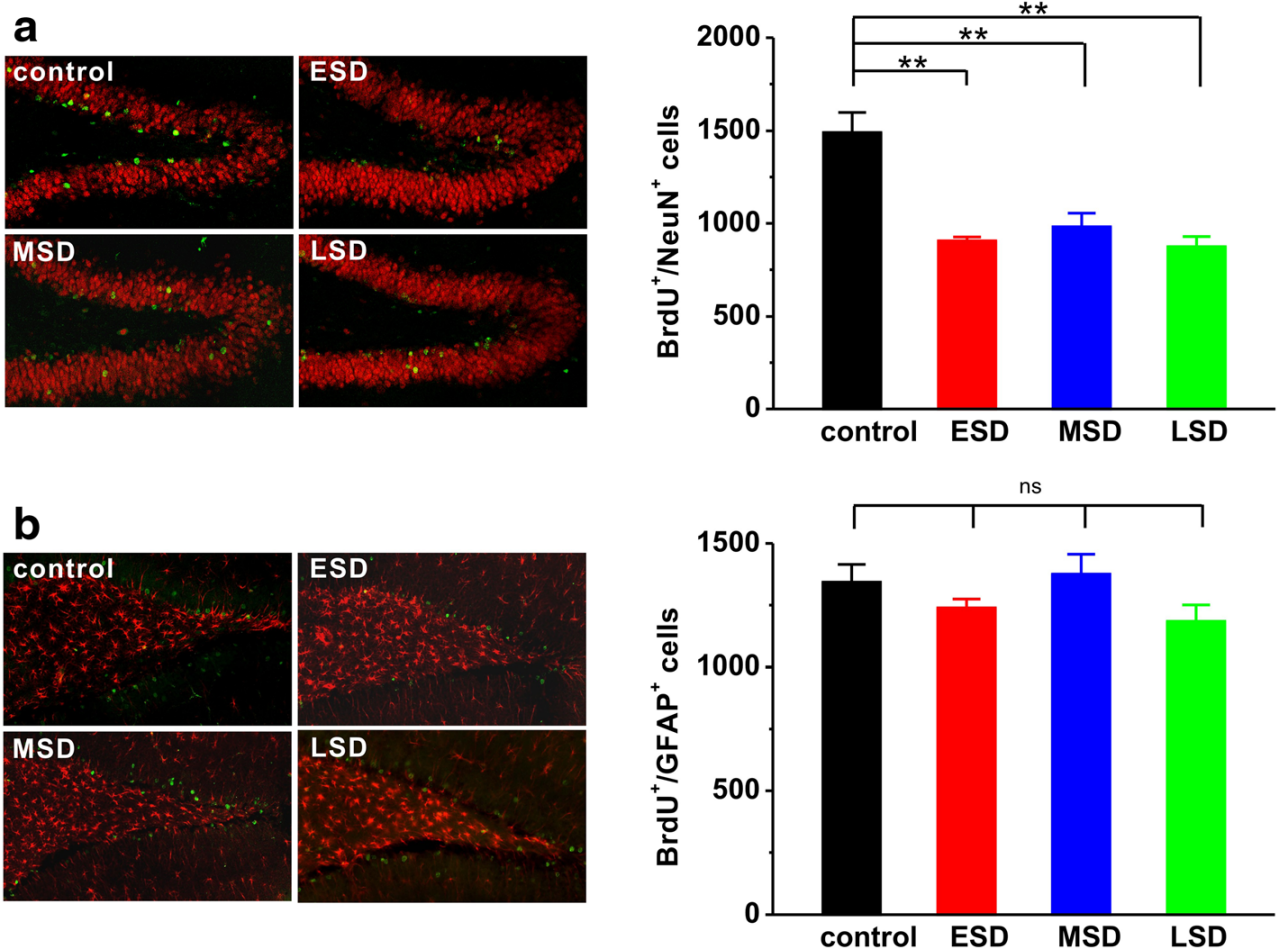
Maternal sleep deprivation reduced adult hippocampal neuronal survival. **a** The offspring of ESD (*n* = 5), MSD (*n* = 5) and LSD (*n* = 5) showed significantly reduced the immunoreactivity (left panel) and number of newborn neuronal phenotype cells (right bar) compared to control (*n* = 5), which were counted 4 weeks after last BrdU administration. **b** No differences were observed in the immunoreactivity (left panel) and number of newborn astrocytic phenotype cells (right bar) among these groups. ^**^ *p* < 0.01 vs. control; ns = no significant difference

In addition, to further determine the survival of newly generated cells and their commitment toward the neuronal lineage, we treated animals with BrdU daily from PND 15–21, and sacrificed the rats 4 weeks after last BrdU administration. The results showed that the number of BrdU-positive cells with neuronal phenotype (F _(3, 16)_ = 16.271, *p* < 0.001; Fig. 6a) rather than astrocytic phenotype (F _(3, 16)_ = 1.833, *p* = 0.182; Fig. 6b) was significantly reduced in the offspring of maternal sleep deprivation compared to the control rats.

Thus, these results suggest that maternal sleep deprivation leads to a significant reduction in adult hippocampal neurogenesis including neuronal proliferation and survival in the offspring rats.

### Maternal sleep deprivation impairs hippocampal LTP and basal synaptic transmission in the offspring rats

**Fig. 7:**
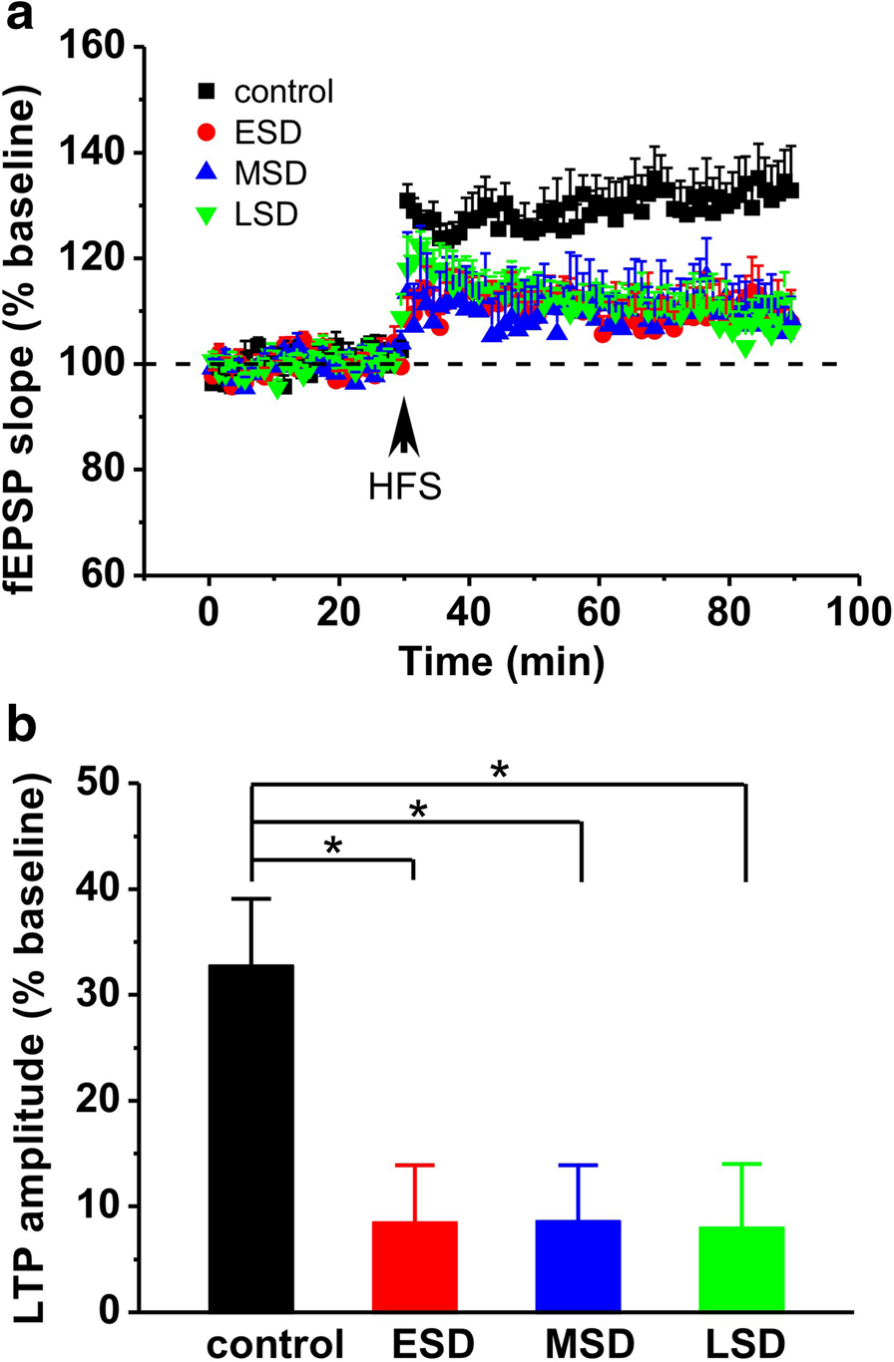
Maternal sleep deprivation impaired hippocampal LTP in vivo. **a** The plots of normalized slopes of fEPSPs showed that HFS (100 Hz for 1 s) induced a much smaller hippocampal LTP in the offspring of ESD (*n* = 6), MSD (*n* = 6) and LSD (*n* = 6) than control (*n* = 5). **b** The bar graph summarized the average percentage change of fEPSP slope immediately before and 55 min after HFS. ^*^ *p* < 0.05 vs. control

**Fig. 8:**
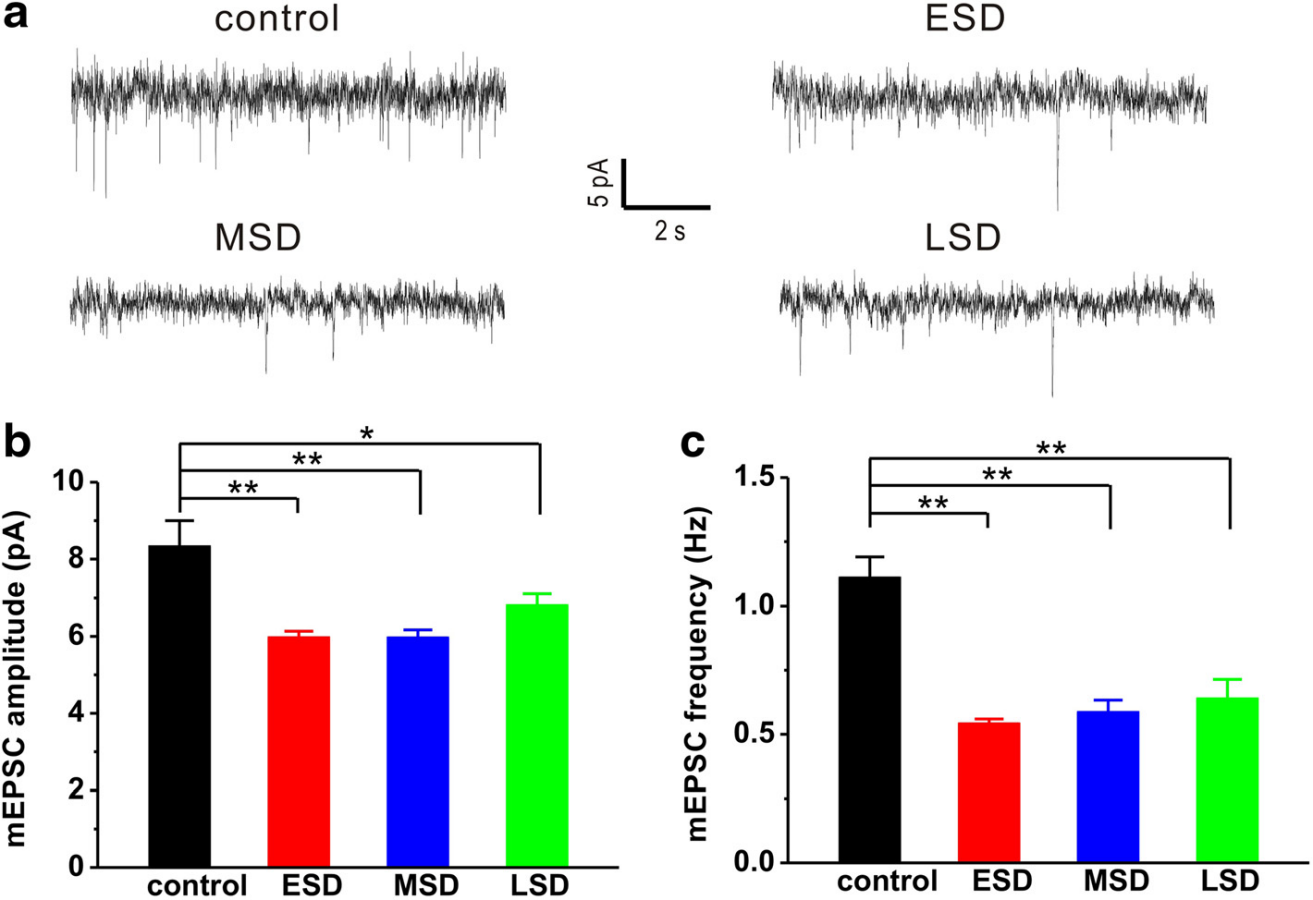
Maternal sleep deprivation reduced excitatory synaptic acticity. The offspring of ESD (*n* = 20), MSD (*n* = 13) and LSD (*n* = 12) showed significantly reduced mEPSC amplitude (**b**) and frequency (**c**) than control (*n* = 14), and corresponding representative traces were shown in graph (**a**). ^*^ *p* < 0.05, ^**^ *p* < 0.01 vs. control

Accumulating evidence supports that hippocampal LTP plays a critical role in learning and memory, and sleep deprivation impairs hippocampal LTP [19, 20]. However, so far there is no study to examine the effect of maternal sleep deprivation on hippocampal LTP induction in the offspring. Thus, we next examined the influence of maternal sleep deprivation on offspring hippocampal LTP. The results showed that HFS induced a reliable LTP in the control rats, whereas the LTP induced by HFS was significantly impaired in the offspring of ESD, MSD and LSD (F _(3, 19)_ = 3.973, *p* = 0.024; Fig. 7a and b). Next, we further investigated the influence of maternal sleep deprivation on excitatory synaptic transmission in the CA1 pyramidal neurons from hippocampal slices. The results showed that both amplitude (F _(3, 55)_ = 8.970, *p* < 0.001; Fig. 8a and b) and frequency (F _(3, 55)_ = 21.487, *p* < 0.001; Fig. 8a and c) of mEPSC were significantly reduced in the offspring of ESD, MSD and LSD compared to the control rats. Altogether, these results indicate that maternal sleep deprivation disrupts hippocampal LTP and excitatory synaptic transmission, thereby may contribute to memory deficits in the offspring rats.

## Discussion

The physiological and biochemical changes of pregnancy may place women at risk for developing specific sleep disorders [3, 4]. Indeed, recent report shows that about two-thirds of the pregnant women are subjected to sleep disorders [25]. Although it is widely accepted that maternal sleep restriction may lead to several harmful consequences to the offspring, human studies on sleep deprivation during pregnancy and the child’s development are difficult and even impossible to be conducted due to ethical and other issues. Therefore, the present study was designed to investigate the effects of maternal sleep deprivation by gentle handling in the rat model on cognitive and emotional functions in the offspring. Gentle handling protocols for sleep deprivation in animal models are widely used in research under laboratory conditions [10, 20], as it is a simple method for sleep deprivation [26].

Previous studies have shown that pregnancy-related hormones such as progesterone, estrogen, cortisol and oxytocin are gradually increased during pregnancy, especially in the third trimester of gestation, which markedly affect sleep quality [27]. Thus, a large number of reports have focused on the influence of sleep deprivation during the last trimester of pregnancy on the development of the offspring [7, 9, 10, 28, 29]. Recently, a report shows that paradoxical sleep deprivation using the multiple platform method at different pregnant period impairs hippocampus-dependent spatial learning and memory in the young offspring rats [6]. In full agreement with this finding, we here reported that maternal sleep deprivation through the gentle handling method at the different stages of pregnancy displayed a similar deficit in spatial learning and memory, especially for the first 3 days of learning in the Morris water maze task (Fig. 1), suggesting a delayed acquisition of spatial memory in the offspring rats. In addition, Gulia and colleagues have recently reported that pups born to sleep deprived mothers displayed a decrease in ultrasonic vocalizations [11], indicating a depressive-like symptom. Consistent with this result, we here found that rats born to mothers undergoing sleep deprivation displayed apparently depression during forced-swimming test (Fig. 2). However, we also found that maternal sleep deprivation significantly induced anxiety-like behaviors in the offspring in both elevated-plus maze and novelty-suppressed feeding tests (Fig. 3 and Fig. 4), which is not consistent with a previous report that sleep deprivation during late pregnancy produces a decrease in anxiety-related behavior, as reflected by hyperactivity and increased risk-taking behavior in the offspring [10]. Given this controversy, further studies are required to determine the exact reason for the different results.

Although our findings, along with evidence accumulated from previous studies [6, 7], reveal that sufficient sleep at any stage of pregnancy is critical for the development of cognitive and emotional functions in the offspring, how maternal sleep deprivation disrupts cognitive and emotional functions in the offspring remains poorly understood. Consistent with recent reports [6, 7], we here confirmed that maternal sleep deprivation resulted in a dramatic decrease in the number of newborn neurons in the DG of the hippocampal formation (Fig. 5 and Fig. 6). Although the function of adult neurogenesis is still under debate, integration of newborn neurons into the existing neuronal circuits may be involved in memory processing and emotional regulation in the hippocampus [30, 31]. Thus, the inhibition of hippocampal neurogenesis induced by maternal sleep deprivation in the present study may contribute to the impairment of hippocampus-dependent spatial learning and memory as well as the increase in depression and anxiety in the offspring. In addition, more recent study has proposed that maternal sleep deprivation may lead to dysregulation in microglial pro- and anti-inflammatory activation, and consequently inhibit adult hippocampal neurogenesis and impair hippocampal-dependent spatial learning and memory, as anti-inflammatory treatment can relief cognitive impairment caused by maternal sleep deprivation in offspring. [7]. Furthermore, activity-dependent hippocampal synaptic plasticity such as LTP has been considered as a cellular mechanism underlying information processing and memory formation [15–17]. We therefore speculate that maternal sleep deprivation may impair hippocampal LTP induction, and subsequently leads to memory deficit in the young offspring rats. Indeed, we here found that both hippocampal LTP (Fig. 7) and basal synaptic transmission (Fig. 8) were significantly suppressed in the young offspring rats born to mothers undergoing sleep deprivation during pregnancy, which may contribute, at least partially, to the disruption of cognitive and emotional functions.

Notably, although we used gentle handling that minimizes stress [32], to perform maternal sleep deprivation, it may still be a stressful procedure. It has been documented that maternal stress produces memory deficits and hippocampal synaptic plasticity impairment in the offspring [33–36]. Thus, the effects of acute stress resulting from sleep deprivation on cognitive and emotional functions in the young offspring rats cannot be occluded in the present study. Additionally, maternal sleep deprivation during their inactive phase may lead to sleep compensation during their active phase. Previous studies have shown that phase-shifting circadian rhythms may influence hippocampus-dependent memory [37, 38]. Thus, maternal sleep deprivation may result in the disruption of circadian rhythms, and subsequently contributes to memory deficits in the offspring.

Although our results show that maternal sleep deprivation suppresses hippocampal neurogenesis and synaptic plasticity that may contribute to memory deficits in the young offspring rats, the exact mechanism still remains unclear. Further research is needed to enhance our understanding of the effect of maternal sleep deprivation on offspring development and the physiological mechanisms underlying this influence.

## Conclusion

Overall, our study shows that maternal sleep deprivation at different stages of pregnancy leads to a significant increase in depression and anxiety, and dramatic deficits in spatial learning and memory. These behavioral changes are associated with the impairments of excitatory synaptic transmission and LTP in the hippocampus.
